# Species delimitation of *Apharyngostrigea* Ciurea, 1927 (Digenea: Diplostomoidea) based on morphology and molecular data from the Neotropical region of Mexico

**DOI:** 10.1017/S0031182025101315

**Published:** 2026-02

**Authors:** Alejandra López-Jiménez, Martín García-Varela, Rogelio Aguilar-Aguilar

**Affiliations:** 1 Departamento de Biología Comparada, Facultad de Ciencias, Universidad Nacional Autónoma de México, Ciudad de México, México; 2 Departamento de Zoología, Instituto de Biología, Universidad Nacional Autónoma de México, Ciudad de México, México

**Keywords:** ABGD, *Apharyngostrigea*, BPP, GMYC, morphology, species delimitation

## Abstract

The genus *Apharyngostrigea* comprises a group of diplostomoidean digeneans that parasitize birds of the family Ardeidae (herons), with approximately 20 species described worldwide. Despite numerous efforts, a robust phylogenetic framework to delimit species within the genus is still lacking, mainly due to the limited morphological variation among its members. This study employed an integrative taxonomic approach, combining nuclear and mitochondrial DNA sequences with morphological data to assess species boundaries within *Apharyngostrigea* based on specimens collected from southeastern Mexico. Using a combination of species discovery (Automatic Barcode Gap Discovery, Assemble Species by Automatic Partition, General Mixed Yule Coalescent and Poisson Tree Processes) and validation methods based on Bayesian gene tree topologies (BPP and PHRAPL). We found high diversity within this genus in southeastern Mexico. Our analyses supported the delimitation of four nominal species that were previously described and validated in this study, along with the redescription of three of them. In addition, through species delimitation methods and morphological examination, we identified two candidate species and/or lineages that require further evidence to be formally described. This study demonstrates that an integrative taxonomic approach provides a robust framework for species delimitation in taxonomically complex groups such as *Apharyngostrigea*.

## Graphical abstract

**Figure:**
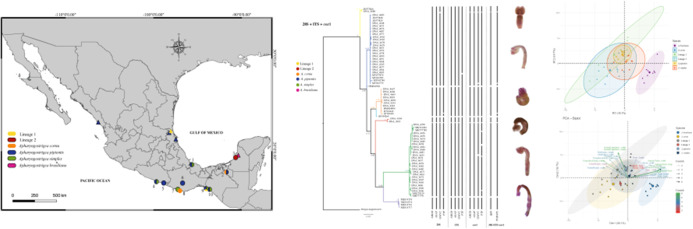

## Introduction

Species delimitation is a practical methodological approach to identify independent evolutionary lineages that lack gene flow among them (Sites and Marshall, 2003; De Queiroz, 2007). In general, species should be delimited objectively and through rigorous analyses. Currently, various methods support an integrative taxonomic approach, especially for analysing taxonomically complex groups (Padial et al., 2010; Carstens et al., 2013). These methods should be complemented with other lines of evidence, such as morphology, behaviour and ecology data (Miralles and Vences, 2013). The main species delimitation methods are subdivided into *de novo* inference methods (without *a priori* defined entities) and validation methods (where predefined entities are tested) (Carstens et al., 2013). These methods can be classified into three main categories: 1) distance-based methods such as Automatic Barcode Gap Discovery (ABGD) (Puillandre et al., 2012) and Assemble Species by Automatic Partition (ASAP) (Puillandre et al., 2021), which analyse pairwise genetic distances among sequences to detect the presence of a barcode gap, 2) network-based methods, such as the REfined Single Linkage (RESL) algorithm, as implemented in the Barcode of Life Data System (BOLD), which employ a graph-based Markov clustering approach to explore connectivity among sequences through random walks in the network (Ratnasingham and Hebert, 2007, 2013) and 3) model-based approaches, such the General Mixed Yule Coalescent (GMYC) model (Pons et al., 2006; Fujisawa and Barraclough, 2013) and Poisson Tree Processes (PTP) model (Zhang et al., 2013; Kapli et al., 2017). These methods apply mixture models with two distinct components, distinguishing within-species and between-species variation. PTP employs two Poisson distributions to model branching events, while GMYC combines a coalescent model with a Yule diversification model (Carstens et al., 2013). Despite the variety of species delimitation methods, few have been applied to the study of parasites, mainly in trematodes (Martínez-Aquino *et al.,* 2013; Herrmann et al., 2014; Locke et al., 2015; Pérez-Ponce de León et al., 2016; Gordy et al., 2017; Pinacho-Pinacho et al., 2018; Vainutis et al., 2023; Fernandez, *et al.,* 2024).

*Apharyngostrigea* Ciurea, 1927 is a cosmopolitan genus of diplostomoidean digeneans that parasitize birds of the family Ardeidae (herons) (Dubois, 1968). Morphologically, members of this genus are characterized by the absence of a pharynx (Niewiadomska, 2002). Currently, the genus comprises 20 species distributed worldwide, associated with ardeids (Dubois, 1938, 1968; Pérez-Vigueras, 1944; Kim et al., 2020). However, the validity of many species within the genus *Apharyngostrigea* remains controversial, mainly due to their high morphological similarity and the lack of molecular data for their corroboration. Particularly in Mexico, two species of *Apharyngostrigea* have been recorded; *A. brasiliana* Szidat, 1929 in the Boat-billed heron (*Cochlearius cochlearius* L.) in Champotón, Campeche from the Gulf of Mexico, and *A. cornu* (Zeder, 1800) Ciurea, 1927 in four ardeids species; Great Egret (*Ardea alba* L.), Green Heron (*Butorides virescens* L.), Black-crowned Night Heron (*Nycticorax nycticorax* L.) and Yellow-crowned Night Heron (*Nyctanassa violacea* L.), in the states of Veracruz, Gulf of Mexico and Sinaloa, Mexican Pacific (Hernández-Mena et al., 2014; López-Jiménez et al., 2022). However, a recent study based on molecular analyses of nuclear and mitochondrial genes suggests that the records of *A. cornu* in Mexico correspond to *A. pipientis* (Faust, 1918) and *Apharyngostrigea* sp., respectively (Locke et al., 2021).

In the present study, we employed an integrative taxonomic approach, combining morphological examination with multiple molecular species delimitation methods to assess species diversity within *Apharyngostrigea* in Mexico. We generated new molecular data based on mitochondrial and nuclear genes and provided additional morphological information for specimens collected in southeastern Mexico.

## Materials and methods

### Specimen collection

**Figure 1.**
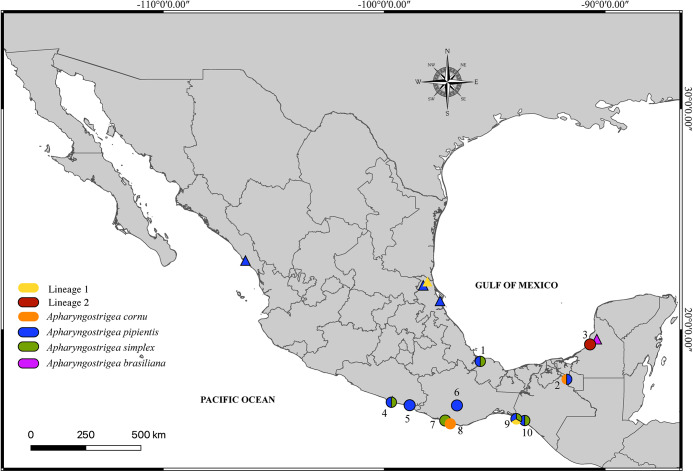
Map of Mexico showing the sampling sites for *Apharyngostrigea* spp. Localities correspond to those listed in Table 1. Sites marked with a triangle indicate those previously sampled by Hernández-Mena et al. (2014) and López-Jiménez et al. (2022).

Birds belonging to the family Ardeidae were collected between 2013 and 2022 from three localities in the Gulf of Mexican slope and seven in the Mexican Pacific slope (Figure 1; Table 1). Ardeids were identified following Howell and Webb (1995), and the American Ornithologist’ Union (1998) guidelines. Adults were obtained from the intestine and placed in Petri dishes with saline solution. The collected digeneans were relaxed using heat-killed distilled water and preserved in 70% ethanol for molecular and morphological analyses.

**Table 1.** Information on the specimens of *Apharyngostrigea* spp. Sampled in this study. Collection sites (CS); sampled localities; geographical coordinates; host names, tree label, and GenBank accession numbers

					GenBank				
CS	Locality	State	Coordinates	Host	Label	ITS	28S	*cox*1	Species/lineages
1	Tlacotalpan	Veracruz	18°37’04.15’’N 95°38’56.10’’W	*Ardea alba*	4248_Pip	PX620579	PX620632	PX642034	*A. pipientis*
					4249_Pip	PX620594	PX620633	PX642035	
				*Butorides* sp.	4250_Sim	PX620599	PX620659	PX642011	*A. simplex*
2	Emiliano Zapata	Tabasco	17°46’29.14’’N 91°44’24.91’’W	*Ardea herodias*	4241_Cor	PX620576	PX620629	PX641999	*A. cornu*
					4242_Cor	PX620577	PX620627	PX642000	
					4893_Cor	PX620574	PX620626	PX642004	
					4894_Cor	PX620575	PX620628	PX642005	
				*Tigrisoma mexicanum*	4908_Pip	PX620593	PX620649	PX642031	*A. pipientis*
3	Champotón	Campeche	19°21’49.9’’N 90°42’56.15’’W	*Egretta thula*	4304_L2	PX620569	PX620630	PX642006	Lineage 2
					4305_L2	PX620570	PX620631	PX642007	
4	Laguna Tres Palos	Guerrero	16°44’35.26’’N 99°42’26.03’’W	*Ardea alba*	4653_Pip	PX620587	PX620634	PX642038	*A. pipientis*
				*Egretta tricolor*	4654_Sim	PX620600	PX620654	PX642013	*A. simplex*
					4655_Sim	PX620619	PX620655	PX642015	
5	Marquelia	Guerrero	16°35’40.88’’N 98°50’37.90’’W	*Nycticorax nycticorax*	4689_Pip	PX620578	PX620639	PX642050	*A. pipientis*
					4690_Pip	PX620591	PX620640	PX642048	
					4691_Pip	PX620592	PX620641	PX642049	
6	Los Ocotes	Oaxaca	16°36’57’’N 96°43’13’’W	*Ardea alba*	4576_Pip	PX620588	PX620642	PX642033	*A. pipientis*
					4577_Pip	PX620583	PX620643	PX642036	
					4578_Pip	PX620589	PX620644	PX642037	
7	Santa María, Cocotepec	Oaxaca	15°48’24.56’’N 97°00’49.79’’W	*Egretta tricolor*	4656_Sim	PX620601	PX620653	PX642010	*A. simplex*
				*Egretta thula*	4657_Sim	PX620602	PX620656	PX642020	*A. simplex*
					4658_Sim	PX620603	PX620657	PX642018	
					4659_Sim	PX620604	PX620669	PX642025	
					4660_Sim	PX620620	PX620670	PX642026	
					4661_Sim	PX620621	PX620671	PX642022	
8	Villa de Tututepec	Oaxaca	15°56’10.98’’N 97°13’38.87’’W	*Ardea herodias*	4667_Cor	PX620571	PX620623	PX642001	*A. cornu*
					4668_Cor	PX620572	PX620624	PX642002	
					4669_Cor	PX620573	PX620625	PX642003	
9	El Zapotal	Chiapas	15°58’20.26’’N 93°51’23.04’’W	*Ardea alba*	4675_Pip	PX620580	PX620650	PX642041	*A. pipientis*
					4676_Pip	PX620581	PX620635	PX642032	
					4677_Pip	PX620595	PX620636	PX642042	
					4678_Pip	PX620586	PX620651	PX642046	
					4679_Pip	PX620598	PX620652	PX642047	
				*Tigrisoma mexicanum*	4687_Pip	PX620582	PX620637	PX642043	*A. pipientis*
				*Egretta tricolor*	4685_Sim	PX620612	PX620665	PX642009	*A. simplex*
					4686_Sim	PX620613	PX620660	PX642023	
					4900_Sim	PX620614	PX620664	PX642030	
				*Egretta thula*	4680_Sim	PX620605	PX620666	PX642014	*A. simplex*
					4681_Sim	PX620610	PX620667	PX642016	
					4682_Sim	PX620611	PX620668	PX642024	
					4683_Sim	PX620617	PX620672	PX642019	
					4684_Sim	PX620618	PX620673	PX642017	
				*Egretta rufescens*	4670_Sim	PX620606	PX620658	PX642008	*A. simplex*
					4671_Sim	PX620607	PX620663	PX642021	
					4672_Sim	PX620608	PX620674	PX642029	
					4674_Sim	PX620609	PX620675	PX642012	
				*Nyctanassa violacea*	4688_L1	PX620590	PX620638	PX642051	Lineage 1
10	Tonalá	Chiapas	15°57’23.63’’N 93°40’36.30’’W	*Butorides virescens*	4902_Pip	PX620584	PX620645	PX642044	*A. pipientis*
					4,9034903_Pip	PX620585	PX620646	PX642045	
				*Ardea alba*	4904_Pip	PX620596	PX620647	PX642040	*A. pipientis*
					4905_Pip	PX620597	PX620648	PX642039	
				*Egretta rufescens*	4906_Sim	PX620615	PX620661	PX642027	*A. simplex*
					4907_Sim	PX620616	PX620662	PX642028	

### Morphological analyses

Some specimens were stained with Mayer’s paracarmine (Merck, Darmstadt, Germany), dehydrated through a graded ethanol series, cleared with methyl salicylate and mounted on permanent slides using Canada balsam. Photographs and measurements were taken with a Leica DM 1000 LED compound microscope (Leica Microsystems CMS GmbH, Wetzlar, Germany). All measurements were recorded in micrometres (μm) and are presented as ranges with the mean in parentheses. Voucher specimens were deposited in the Colección Nacional de Helmintos (CNHE), Instituto de Biología, Universidad Nacional Autónoma de México (UNAM), Mexico City.

### Molecular data

To obtain genomic DNA, preserved samples were digested individually in tubes and digested overnight at 56°C in a solution containing 10 mM Tris–HCl (pH 7·6), 20 mM NaCl, 100 mM Na_2_ EDTA (pH 8·0), 1% sarkosyl and 0·1 mg mL^-1^ proteinase K. Following digestion, DNA was extracted from the supernatant using the DNAzol reagent (Molecular Research Center, Cincinnati, OH, USA) according to the manufacturer’s instructions. The internal transcribed spacers (ITS1-5.8s-ITS2) of the nuclear ribosomal DNA were amplified using the forward primer BD1 (5′−GTC GTA ACA AGG TTT CCG TA−3′) and the reverse primer BD2 (5′−ATC TAG ACC GGA CTA GGC TGT G−3′) (Bowles et al., 1995). Partial fragments of domains D1–D3 of the large subunit of nuclear ribosomal DNA (28S) were amplified using the forward primer 391 (5′−AGCGGAGGAAAAGAAACTAA−3′) (Nadler et al., 2000) and the reverse primer 536 (5′−CAGCTATCCTGAGGGAAAC−3′) (García-Varela and Nadler, 2005). A fragment of *cox*1, approximately 470 bp in length, was amplified using the forward primer AphaF (5′−TATGATTTTTTTYTTTTTRATG−3′) and the reverse primer AphaR (5′−CCAAACYAACACMGACAT−3′) (see López-Jiménez et al., 2023). Polymerase chain reactions (PCRs) were carried out in 25 μL reaction volumes following the manufacturer’s instructions. The PCR cycling conditions included an initial denaturation at 94°C for 3 min, followed by 35 cycles of 94°C for 1 min, annealing at 50°C for 28S and ITS and 55°C for *cox*1 for 1 min, and extension at 72°C for 1 min, with a final post-amplification incubation at 72°C for 10 min. Sequencing reactions were performed using ABI Big Dye terminator sequencing chemistry (Applied Biosystems, Boston, MA, USA), and the reaction products were separated and detected using an ABI 3730 capillary DNA sequencer. Contigs were assembled, and base-calling differences were resolved using CodonCode Aligner v.12.0.1 (CodonCode Corporation, Dedham, MA, USA).

### Alignment and phylogenetic analyses

The new sequences were aligned with those of other species of the genus *Apharyngostrigea* available in GenBank (Supplementary material S1). Matrices for each gene were aligned individually using the ClustalW algorithm (Thompson et al., 1997) with the default parameters in MEGA v.11 software (Tamura et al., 2021). Nucleotide substitution models for each molecular marker were selected using JModelTest v.2.1.10 (Darriba et al., 2012), applying the optimal Akaike information criterion (AIC) (Akaike, 1974). The selected models were HKY + I for the 28S gene, TVM + G for the ITS gene and T1M1 + I + G for the *cox*1 gene. Individual gene trees and the concatenated dataset (28S, ITS and *cox*1) were analysed using maximum likelihood (ML) and Bayesian inference (BI). The species *Strigea magnirostris (*López-Jiménez et al., 2023) sequences were used as outgroup to root the trees in all analyses (both individual and concatenated datasets). For ML analyses, we used the software RAXML v.8.2.12 (Stamatakis, 2006), to generate gene trees based on the substitution model closest to previous estimates, with 1,000 replicates, using the computational resource Cyberinfrastructure for Phylogenetic Research Science Gateway v3.3 (Miller et al., 2010). Bayesian inference analyses for individual and concatenated trees (28S, ITS and *cox*1) were conducting using MrBayes v3.2.6 (Ronquist et al., 2012), with Markov chain Monte Carlo (MCMC) simulations run for 10 million generations, sampling every 1,000 generations and discarding the first 2,500 samples as ‘burn-in’ (25%). The results were visualized using FigTree v1.4.2 (Rambaut, 2012). Additionally, we generated individual gene trees under a molecular clock framework to perform the GMYC analysis using BEAST v.2.7.7 (Suchard et al., 2018). The analysis was conducted under a Yule model and a coalescent model with a constant population size, using both constant and relaxed molecular clocks. A total of 10,000 replicates were run, ensuring that all output parameters had an Effective Sample Size (ESS) >200.

### Species delimitation (discovery and validation)

Species delimitation was performed with two different approaches, following the methodology of Carstens et al. (2013). First, four exploratory or discovery methods were applied to identify potential candidate species based on *a priori* information using single-gene data (28S, ITS and *cox*1). Two distance-based methods were employed: ABGD (Puillandre et al., 2012) with the following parameters–Pmin: 0·01, Pmax:0·1, Steps: 10, Nb bins: 20 and Jukes-Cantor distances (JC69)–and Assemble Species by Automatic Partitioning (ASAP) (Puillandre et al., 2021), which was run with 1,000 replicates under the JC69 genetic distance model. Additionally, two model-based approaches, General Mixed Yule Coalescent model (GMYC) (Pons et al., 2006) and Poisson Tree Processes model (PTP) (Zhang et al., 2013), were applied. The trees with Yule clock, coalescent constant and relaxed clock were generated in BEAUTi and BEAST v.2.7.7 (Drummond et al., 2012) executed for at least 20 million MCMC generations, sampling every 10,000 generations. Convergence of the two chains was assessed using TRACER v.1.7 (Rambaut et al., 2018). GMYC analyses were conducted in R v.4.1.3 (Allaire, 2012) with the ‘*splits*’ package (Ezard et al., 2021) for single and multiple threshold GMYC. PTP analyses were executed using the web server (https://species.h-its.org/) (Zhang et al., 2013) with default settings: rooted tree, MCMC generations = 100,000, burn-in = 0·1, seed = 123 and thinning = 100.

Candidate species were assessed using two species validation methods based on phylogenetic trees constructed from multilocus data: BPP and PHRAPL. Bayesian species delimitation was executed using BPP (Bayesian Phylogenetics and Phylogeography) v.4.3.8 (Yang, 2015), a Bayesian Markov chain Monte Carlo (MCMC) program designed to analyse multilocus sequence data under the multispecies coalescent (MSC) model. BPP requires sequence data as input and a predefined guide tree topology (Yang and Rannala, 2014). It can be used for four types of inference problems (Yang, 2015). We conducted an A11 analysis (species delimitation = 1 and species tree = 1): which jointly estimates species delimitation/assignment and species tree models (Yang and Rannala, 2014). The analysis was run for 100,0000 rjMCMC generations with burn-in = 8,000 and sampling frequency of 2. Additionally, Phylogeographic Inference Using Approximate Likelihoods (PHRAPL) was employed to evaluate alternative demographic and evolutionary scenarios underlying the observed genetic patterns. The analysis was conducted using ML gene trees within the PHRAPL framework, implemented in R v.4.1.3 with the ‘*phrapl*’ package (Jackson et al., 2017). Four subsamples per gene tree were used, and the grid search was performed by evaluating 10,000 simulated trees. The five best-fitting models were selected based on their AIC values.

### Analysis of morphometric data

For these species recognized through the previous delimitation methods, we conducted a principal component analysis (PCA) to explore and describe the patterns of morphological variation in *Apharyngostrigea* specimens found in Mexico. A total of 24 morphometrics variables were considered from 47 specimens belonging to four species – *A. cornu* (*n* = 10)*, A. pipientis* (*n* = 10)*, A. simplex* (*n* = 8) and *A. brasiliana* (*n* = 10) (measurements obtained by López-Jiménez et al., 2022) – as well as two undescribed species, Lineage 1 (*n* = 7) and Lineage 2 (*n* = 2). The PCA was performed using the ggplot2, ggfortify, cluster, lfda and reader packages implemented in R v.4.1.3 (R Core Team, 2022) (Supplementary Material S2).

## Results

### Phylogenetic analyses and species boundaries

**Figure 2.**
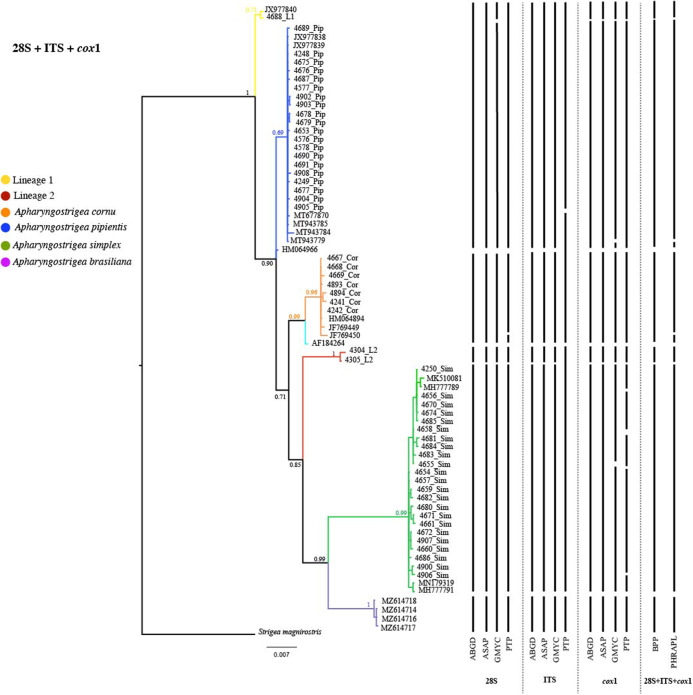
Results of phylogenetic analysis and species delimitation. Bayesian majority rule (50%) phylograms based on the concatenated (28S + ITS + *cox*1) gene sequences of *Apharyngostrigea*. Black vertical lines indicate that the corresponding genes in the phylogram and their concatenated sequences were recovered by the different species delimitation methods employed: Automatic Barcode Gap Discovery (ABGD); Assemble Species by Automatic Partitioning (ASAP); Generalized Mixed Yule Coalescent method (GMYC); Poisson Tree Processes (PTP); Bayesian Phylogenetics and Phylogeography (BPP); Phylogeographic Inference Using Approximate Likelihoods (PHRAPL).

Phylogenetic analyses were performed for each dataset individually and for the concatenated dataset. The 28S alignment included 60 sequences with 1,157 characters, the ITS dataset included 64 sequences with 1,057 characters and the *cox*1 dataset included 72 sequences with 371 characters. The concatenated tree of the three genes (28S + ITS + *cox*1), including a total of 174 individuals (the same individuals for which all three markers were obtained) (Table 1). These sequences were analysed with other species of *Apharyngostrigea* available in GenBank (Supplementary Material S1). Trees for individual markers are shown in Supplementary Material S3. In general, the individual trees for each marker showed the same topology, highly supported by posterior probability values. Phylogenetic hypotheses generated through Bayesian analyses of the concatenated sequences (28S + ITS + *cox*1), as well as results from the species delimitation analyses, are shown in Figure 2. Overall, these analyses recognized four nominal species and two candidate species and/or lineages in Mexico (Figure 2). The first clade consisted of four sequences of *A. brasiliana* (MZ614714, MZ614716–18) collected from the Boat-billed heron in a single locality from the Gulf of Mexico slope. These sequences formed a sister clade with 26 sequences of *Apharyngostrigea*, including four sequences identified as *A. simplex* (MK510081, MH777791, MH777789, MN179319) from Brazil and Argentina (López-Hernández et al., 2019; Locke et al., 2021). The third clade consisted of two sequences of *Apharyngostrigea* sp. (Lineage 2) collected from the Snowy egret (*Egretta thula* Molina) in Champotón, Campeche, Gulf of Mexico in the present study. The fourth clade contained seven sequences collected from the Great blue heron (*Ardea herodias* L.) in two localities, Emiliano Zapata, Tabasco and Villa Tututepec, Oaxaca, from Mexico. This clade was nested with sequences identified as *A. cornu* (HM064894, JF769449–50, AF184264) from Canada and Ukraine (Tkach et al., 2001; Locke et al., 2011). The fifth clade contained two sequences originally identified as *A. cornu* from Mexico and reassigned as *A. pipientis* by Locke et al. (2021) (JX977838–39), along with sequences identified as *A. pipientis* (MT677870, MT943784–85 and MT943779) from Argentina and Canada (Locke et al., 2021). Finally, the sixth clade contained two sequences identified as *Apharyngostrigea* sp. (Lineage 1) from the Yellow-crowned Night Heron (*N. violacea*) collected previously in the Cortadura, Veracruz by Hernández-Mena et al. (2014), in the Gulf of Mexico and Zapotal, Chiapas, Mexican Pacific, in the present study.

Species discovery methods based on mitochondrial data tend to estimate a higher number of species compared to those using nuclear genes (such as 28S and ITS), due to the lower genetic variability observed in nuclear markers. For instance, within the *A. simplex* clade, species delimitation varied depending on the method used: ABGD and ASAP grouped it as a single species, whereas GMYC and PTP methods recognized two and five distinct species, respectively. In contrast, validation methods such as BPP and PHRAPL consistently supported the presence of four nominal species (*A. cornu, A. pipientis, A. simplex* and *A. brasiliana*) along with two candidate species and/or lineages were also recovered in Mexico with high posterior probability support values (see Figure 2). Overall, most species delimitation approaches (discovery and validation), whether based on single-locus or multilocus analyses, recognized four nominal species and two candidate species and/or lineages within the genus *Apharyngostrigea* (Figure 2).

### Morphological differentiation

**Figure 3.**
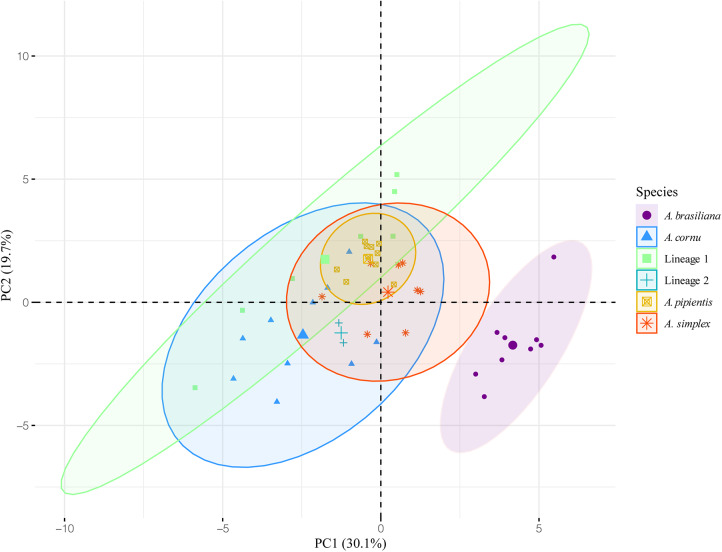
Principal component analyses (PCA) of four species and two lineages of *Apharyngostrigea* were conducted with 24 variables from 47 individuals.

**Figure 4.**
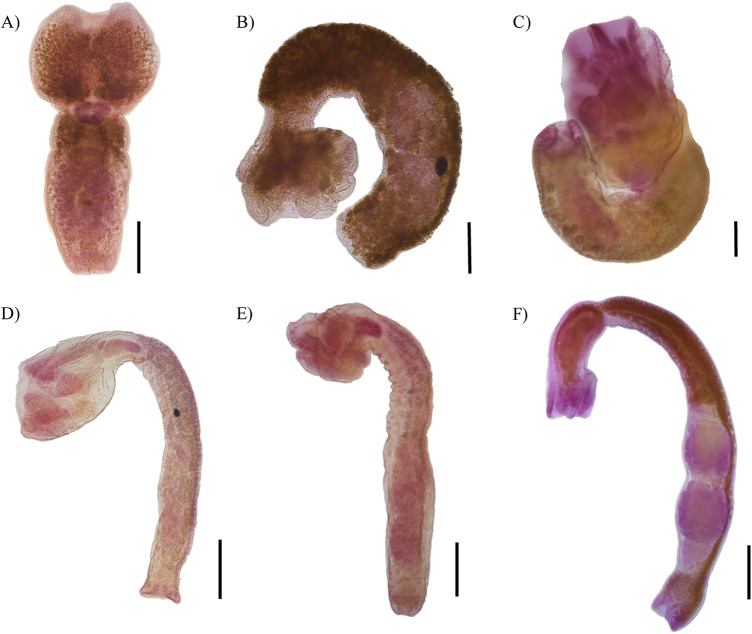
Photographs of specimens from analysed lineages and species of *Apharyngostrigea*. (A) Lineage 1. Scale bars = 500 μm. (B) Lineage 2. Scale bars = 200 μm. (C) *A. cornu.* Scale bars = 200 μm. (D) *A. pipientis*. Scale bars = 500 μm. (E) *A. simplex*. Scale bars = 500 μm. (F) *A. brasiliana*. Scale bars = 500 μm.

PCA was conducted to corroborate the morphological differences between the species of *Apharyngostrigea* found in the present study. Morphometric data were obtained from 47 specimens corresponding to four species – *A. pipientis, A. cornu, A. simplex* and *A. brasiliana*–as well as two undescribed species (Lineage 1 and Lineage 2). The combined, simultaneous analysis of all considered groups does not enable a distinction between the entities, due to the conservative morphology of the genus. An exception was observed in the morphometrical data of *A. brasiliana*, which formed a distinct cluster separate from other species (Figures 3 and 4). Based on cumulative variance and eigenvalues, the first five principal components (PCs) were retained for at least 80% of total variation. However, only PC1 and PC2 were statistically significant. According to the loading values, a combination of 10 variables contributed significantly to PC1, which explained 30·1% of the total variation (95% confidence interval; *P* < 0·001), while a combination of eight variables contributed significantly to PC2, explaining 19·7% of the total variation (95% confidence interval; *P* < 0·001) (Supplementary Material S4).

### Morphological redescription

*Apharyngostrigea cornu* (Zeder, 1800) Ciurea, 1927

*Host: Ardea herodias* Linnaeus, 1758 (Pelecaniformes: Ardeidae)

*Locality*: Emiliano Zapata, Tabasco, Mexico (17°46’29.14’’N, 91°44’24.91’’W)

*Site of infection*: Intestine.

*Voucher material*: CNHE 12482

*GenBank accession number*: 28S: PX620623–629; ITS: PX620571–577; *cox*1: PX641999–2005.

Redescription based on 10 gravid adults (Figure 4C; Table 2). Body distinctly bipartite, 1846–2529 (2299) in total length. Tegument smooth. Forebody caliciform or pyriform, slightly wider than long, 578–750 × 605–809. Ratio of forebody length to body length: 1: 3·1–4·2 (3·5). Hindbody cylindrical, longer than forebody, 1268–1906 × 342–530. Ratio of forebody length to hindbody length: 1: 2·1–3·2 (2·5). Oral sucker subterminal, 147–183 × 120–160. Ventral sucker oval, larger than oral sucker, 230–258 × 148–300. Suckers width ratio: 1: 1·05–1·93 (1·44). Pharynx absent. Holdfast organ has well-developed dorsal and ventral lips. Proteolytic gland well-developed 183–257 × 119–270, situated in the intersegmental region. Testes in tandem are deeply multilobed, and are situated in the second third of the hindbody. Anterior testis 212–303 × 186–265, posterior testis slightly larger than anterior testis 249–316 × 232–289. Seminal vesicle sinuous, postesticular. Ovary oval or reniform, pretesticular 108–177 × 207–235, situated in 27–31/100 of hindbody. Mehlis’ gland and vitelline reservoir in intertesticular region. Vitelline follicles are distributed in both regions of the body, being scarce in the forebody, where they penetrate into the lobes of the holdfast organ, while in the hindbody, they are concentrated in the neck region (preovarian zone), extending dorsally toward the testes and reaching the vesicle seminal or copulatory bursa. Copulatory bursa well developed, 218–336 × 332–491. Genital cone small, 146–238 × 170–246. Relatively few eggs (2–10), oval 55–91 × 45–70. Excretory pore terminal.

**Table 2.** Comparative measurements of adult specimens of *Apharyngostrigea cornu* (Zeder, 1800) Ciurea, 1927

Host	*Ardea herodias*	*Ardea cinerea*	*Nycticorax nycticorax Ardea herodias*	Ardeidae
Locality	Emiliano Zapata, Tabasco, Mexico	Europe	Minnesota, USA	Europe, Africa
Source	Present study	Dubois, (1938)	Olsen, (1940)	Dubois, (1968)
Body length (BL)	1,846–2,529 (2,299)	2,000–5,700	2,800–4,000	Up to 6,300
Forebody length	578–750 (641)	600–1,300	766–1,330	600–2,200
Forebody width	605–809 (717)	650–1,380	633–800	630–1,700
Hindbody length	1,268–1,906 (1,658)	1,400–4,500	1,760–2,950	1,400–4,500
Hindbody width	342–530 (423)	510–970	266–650	270–1,320
HL/FL	2·1–3·2 (2·5)	2·0–5·2	3·6^a^	1·6–5·2
Oral sucker length	147–183 (167)	100–200	124–208	100–260
Oral sucker width	120–160 (141)	95–190	76–164	95–260
Ventral sucker length	230–258 (238)	180–300	132–300	180–340
Ventral sucker width	148–300 (204)	180–235	120–280	180–360
Proteolytic gland length	183–257 (215)	300–360	240–333	160–450
Proteolytic gland width	119–270 (173)	225–270	160–266	120–350
Ovary length	108–177 (135)	200–360	124–400	100–460
Ovary width	207–235 (216)	150–240	180–483	150–640
Ovary position in HB	27–31/100	–	Middle	27–49/100
Anterior testes length	212–303 (252)	390–660	472^a^	130–720
Anterior testes width	186–265 (241)	405–900	333^a^	220–1,160
Posterior testes length	249–316 (289)	400–720	361^a^	160–880
Posterior testes width	232–289 (261)	360–990	388^a^	220–1,200
Copulatory bursa length	218–336 (262)	–	333^a^	270–330
Copulatory bursa width	332–491 (406)	–	361^a^	–
Genital cone length	146–238 (199)	300–315	194^a^	240–530
Genital cone width	170–246 (199)	180–270	250^a^	180–510
Eggs (*N*)	2–10	–	21–25^a^	Few
Egg length	55–91	80–110	111^a^	80–118
Egg width	45–70	54–68	55^a^	50–75

a Estimated from Figures 3 and 4 in Olsen, 1940.

#### Remarks

*Apharyngostrigea cornu* was originally described as *Distoma cornu* by Zeder (1800), based on specimens found in the intestine of the Grey Heron (*Ardea cinerea* L.) in Europe. Later, Rudolphi (1809, 1819) transferred it to the genus *Amphistoma* and subsequently renamed it *Monostoma cornu*, respectively. Over time, this taxonomic classification became the subject of debate (see Dubois, 1938, 1968). A detailed morphological study of the species was conducted by Ciurea (1927). Morphologically, *A. cornu* is distinguished from its congeners by the presence of abundant vitelline follicles in both parts of the body, a relatively large body size and an ovary situated between 27 and 49/100 of the hindbody (Dubois, 1968). However, our specimens collected from the intestine of the Great Blue Heron (*A. herodias*) from Emiliano Zapata, Tabasco, in the Neotropical region of Mexico showed a reduced number of vitelline follicles in the forebody compared to previous descriptions (Figure 4C). Additionally, our specimens exhibited some degree of intraspecific morphological variation. For example, they presented lower values in the following characteristics: anterior testes width (186–265 *vs* 220–1160), genital cone length (146–238 *vs* 194–530) and egg size (55–91 × 45–70 *vs* 80–118 × 50–75) (see Table 2). *A. cornu* has been reported parasitizing primarily ardeid birds in Europe and Africa (Dubois, 1938, 1968; Richard, 1964; Olson et al., 2003). In North America, adult specimens of *A. cornu* have been recorded in *A. herodias* and *N. nycticorax* from Canada and the United States. Additionally, the metacercariae (larval form) have been reported in cyprinid fish such as *Catostomus commersonii* Lacepède, *Notemigonus crysoleucas* Mitchill and *Pimephales notatus* Rafinesque in Canada (Olsen, 1940; Locke et al., 2011).

*Apharyngostrigea pipientis* (Faust, 1918)

*Host: Ardea alba* Linnaeus, 1758 (Pelecaniformes: Ardeidae)

*Locality*: Zapotal, Chiapas, Mexico (15°58’20.26’’N, 93°51’23.04’’W).

*Site of infection*: Intestine

*Voucher material*: CNHE 12483

*GenBank accession number*: 28S: PX620632–637, PX620639–652; ITS: PX620578–589, PX620591–98; *cox*1: PX642031–050

Redescription based on 10 gravid adults (Figure 4D; Table 3). Body virguliform, 2·4–3·4 mm (2·84 mm) in total length. Tegument smooth. Forebody with median opening, 601–902 × 649–877. Ratio of forebody length to body length: 1: 3·4–4·7 (3·9). Hindbody recurved, longer than forebody 1766–2523 × 321–516, with maximum width in testicular zone. Ratio of forebody length to hindbody length: 1: 2·4–3·7 (2·9). Oral sucker subterminal, well developed, 112–170 × 102–157. Ventral sucker, larger than oral sucker 195–256 × 152–199. Pharynx absent. Holdfast organ well-developed dorsal and ventral lips. Proteolytic gland oblongue 231–377 × 121–191, located in the intersegmental region. Testes in tandem multilobed, located in second or third of hindbody. Anterior testis 190–360 × 150–251, posterior testis 196–422 × 160–229. Seminal vesicle long, postesticular. Ovary reniform, pretesticular 154–243 × 120–175, situated approximately 22–48/100 of hindbody. Mehlis’ gland intertesticular. Vitelline follicles occupy the entire length of the hindbody, densely concentrated in the preovarian region, extending dorsally to the testes and reaching the end of the body, with a lower density in the forebody, penetrating into the lobes of the holdfast organ. Copulatory bursa large, 281–388 × 240–364. Genital cone small, 174–224 × 145–228, genital atrium with a small opening. Eggs numerous 13–60 (30), oval 50–88 × 38–53. Excretory pore ventro-subterminal.

**Table 3.** Comparative measurements of adult specimens of *Apharyngostrigea pipientis* (Faust, 1918)

Host	*Ardea alba*	*Pigeon domestique* (exp.)	*Botaurus lentiginosus*	Ardeidae	*Ardea cinerea jouyi, E. intermedia, N. nycticorax*	*Ardea alba egretta, Ardea herodias, Botaurus lentiginosus, Nycticorax nycticorax*
Locality	Zapotal, Chiapas, Mexico	Michigan, USA	Wisconsin and Michigan, USA		Korea	Formosa, Argentina; Quebec, Canada
Source	Present study	Olivier (1940)	Dubois and Rausch (1950)	Dubois (1968)	Kim et al. (2020)	Locke et al. (2021)
Body length (BL)	2,436−3,407 (2,848)	1,650–1,950^a^	2,100–3,650	3,650	4,100–4,800	1,575–4,000 (2,906)
Forebody length	601–902 (727)	420–500	370–750	370–750	1,000–1,200	650–1,000 (823)
Forebody width	649–877 (753)	340–470	390–560	340–720	800–900	476–677 (577)
Hindbody length	1,766–2,523 (2,121)	1,230–1,450	1,740–3,150	1,100–3,150	3,200–3,600	850–3,000 (2,074)
Hindbody width	321–516 (387)	230–280	300–480	230–610	430–460	300–551 (412)
HL/FL	2·4–3·7 (2·9)	3·9^a^	3·6–6·3	1·7–6·3	2·7–3·7^a^	1·2–3·2 (2·5)
Oral sucker length	112–170 (134)	–	96–110	96–140	104–132 (diam.)	103–153 (134)
Oral sucker width	102–157 (121)	70–90	80–90	70–125	–	50–174 (99)
Ventral sucker length	195–256 (220)	–	120–190	120–215	185–214 (diam.)	120–251 (166)
Ventral sucker width	152–199 (176)	–	110–145	110–195	–	104–172 (143)
Proteolytic gland length	231–377 (275)	110–130	215–315	110–340	353	121–320 (212)
Proteolytic gland width	121–191 (152)	–	155–200	110–200	177	183–270 (222)
Ovary length	154–243 (199)	120–160	165–250	80–190	260	160
Ovary width	120–175 (150)	90–100	110–190	120–250	96	87
Ovary position in HB	22–48/100	33–38/100^a^	30–47/100	30–47/100	–	50/100
Anterior testes length	190–360 (256)	150–190	315–530	150–530	130	238–338 (294)
Anterior testes width	150–251 (200)	130–140	200–320	130–320	260	222–436 (291)
Posterior testes length	196–422 (311)	150–200	340–630	150–630	174	238–413 (332)
Posterior testes width	160–229 (190)	130–140	200–320	130–320	243	160–436 (283)
Copulatory bursa width	240–364 (327)	–	–	–	–	260–464 (359)
Genital cone length	174–224 (202)	–	–	150–220	–	–
Genital cone width	145–228 (195)	–	–	110–210	–	–
Eggs (*N*)	13–60	6–11^a^	–	Few to many	Numerous	0–40 (12)
Egg length	50–88 (69)	–	85–95	78–95	92	64–103 (85)
Egg width	38–53 (45)	–	52–70	47–70	62	45–60 (54)

a Measurements estimated by Locke et al. (2021).

#### Remarks

*Apharyngostrigea pipientis* was described by Olivier (1940) after experimentally infecting a non-natural host (*Pigeon domestique* Gmelin) in Michigan, USA. Since then, it has been reported in North and Central America and has been referred to with different names (Dubois and Rausch, 1950; Dubois, 1968). Morphologically, *A. pipientis* is distinguished from its congeners by having an oblong proteolytic gland situated in the upper portion of the hindbody, as well as its virguliform body shape (Dubois, 1968). Our specimens collected from the intestine of the Great Egret (*A. alba*) in the locality Zapotal, Chiapas, from the Neotropical region of Mexico, are morphologically similar to *A. pipientis* from previous studies (Figure 4D; Table 3). However, our specimens do not have spines on the tegument as previously reported by Locke et al. (2021). *A. pipientis* has been mainly reported parasitizing ardeid birds in the Americas, such as *Botaurus lentiginosus* Rackett*, B. virescens, A. alba egretta, A. herodias, Ixobrychus exilis* Gmelin and *N. nycticorax* from Canada, USA, Cuba and Argentina (Pérez-Vigueras, 1944; Dubois and Rausch, 1950; Locke et al., 2021).

*Apharyngostrigea simplex* (Johnston, 1904) Szidat, 1929

*Host: Egretta thula* Molina, 1782 (Pelecaniformes: Ardeidae)

*Locality*: Santa María Cocotepec, Oaxaca, Mexico (15°48’24.56’’N, 97°00’49.79’’W).

*Site of infection*: Intestine.

*Voucher material*: CNHE 12484

*GenBank accession number*: 28S: PX620653–675; ITS: PX620599–621; *cox*1: PX642008–030

Redescription based on eight gravid adults (Figure 4E; Table 4). Body distinctly bipartite, 2·4–3·4 mm (3·01 mm) in total length. Tegument smooth. Forebody bulbiform, 577–766 × 586–835. Hindbody claviform, longer than forebody 1863–2844 × 344–515. Ratio of forebody length to hindbody length: 1: 2·6–4·6 (3·4). Oral sucker subterminal well developed, 107–164 × 114–129. Ventral sucker oval, larger than oral sucker, 175–242 × 135–180. Pharynx absent. Proteolytic gland is large, 186–266 × 130–178, situated in intersegmental region. Testes in tandem, multilobed. Anterior testis 218–389 × 197–381, posterior testis slightly longer than anterior testes 260–418 × 270–357. Seminal vesicle long, postesticular. Ovary ovoid, pretesticular 119–182 × 100–140, situated approximately 33–48/100 of hindbody. Mehlis’ gland and vitelline reservoir in intertesticular region. Vitelline follicles are densely concentrated in the preovarian region of the hindbody, extending to the posterior margin of the posterior testis; in the forebody, vitelline follicles are sparse and scattered around the with sucker ventral. Copulatory bursa poorly delimited, 227–327 × 222–383, with a slightly developed muscular ring (*Ringnapf*). Genital cone small, covered with minute spines 128–229 × 141–238. The ejaculatory duct and uterus converge at the base of the genital cone to form the hermaphroditic duct. The genital atrium has a large opening. Uterus with 4–11 (7) eggs, oval 67–97 × 42–66. Excretory pore subterminal.

**Table 4.** Comparative measurements of adults *Apharyngostrigea simplex* (Johnston, 1904)

Host	*Egretta thula*	*E. novaehollandiae E. garzetta Ardea plumifera*	*E. thula*	*E. thula*
Locality	Sta. María Cocotepec, Oaxaca, Mexico	Australia	Buenos Aires, Argentina	Daireux, Argentina
Source	Present study	Dubois and Pearson, (1965)	Ostrowski de Núñez, (1989)	Locke et al. (2021)
Body length (BL)	2,440–3,460 (3,017)	3,800	2,017–4,881 (2,823)	2,269–2,369 (2,319)
Forebody length	577–766 (677)	640–950	462–1,215 (690)	469–629 (549)
Forebody width	586–835 (724)	370–780	364–1,045 (593)	600–638 (619)
Hindbody length	1,863–2,844 (2,340)	1,540–3,090	1,290–3,666 (2,153)	1,741–1,800 (1,770)
Hindbody width	344–515 (432)	320–700	316–972 (525)	425–459 (442)
HL/FL	2·6–4·6 (3·4)	1·6–4·3 (2·9)	2·2–6·9 (5·7)	2·8–3·8 (3·3)
Oral sucker length	107–164 (136)	110–165	63–185 (129)	69
Oral sucker width	114–129 (120)	90–110	71–168 (113)	71
Ventral sucker length	175–242 (215)	150–200	126–268 (191)	119–169 (144)
Ventral sucker width	135–180 (158)	130–157	134–252 (186)	145–167 (156)
Proteolytic gland length	186–266 (219)	250–400	151–378 (261)	145
Proteolytic gland width	130–178 (155)	110–220	117–252 (175)	217
Ovary length	119–182 (147)	110–260	101–268 (147)	102–130 (116)
Ovary width	100–140 (122)	145–340	84–412 (182)	145–179 (162)
Ovary position in HB	33–48/100	41–56/100	Near midline	39–47 (43%)
Anterior testes length	218–389 (304)	190–530	269–630 (374)	251
Anterior testes width	197–381 (268)	200–420	210–840 (384)	242
Posterior testes length	260–418 (350)	180–490	252–630 (387)	338
Posterior testes width	270–357 (311)	200–420	252–840 (393)	251
Genital cone length	128–229 (172)	160–260	–	145–266 (205)
Genital cone width	141–238 (201)	130–200	–	121–126 (123)
Eggs (*N*)	4–11 (7)	Numerous	9–100	8–15 (12)
Egg length	67–97 (83)	94–110	90–110	86–95 (92)
Egg width	42–66 (52)	57–68	50–70	52–55 (54)

#### Remarks

*Apharyngostrigea simplex* was originally described by Johnston (1904) from the host *Egretta novaehollandiae* Latham in Australia. Later, Dubois and Pearson (1965) provided a redescription based on individuals collected from other ardeids (*E. garzetta* and *A. plumifera*) in Australia. Our specimens, collected from *E. thula* in the Neotropical region of Mexico, are similar to those described by Ostrowski de Núñez (1989) and Locke et al. (2021) from the same host species in Argentina (Figure 4E, Table 4). However, Locke et al. (2021) reported the presence of tegumental spines, which in our specimens were observed exclusively on the genital cone, exhibiting a distinct pattern of spination.

## Discussion

Species delimitation is a growing area of research in systematic biology (Sites and Marshall, 2003; Camargo and Sites, 2013; Flot, 2015). This approach is based on the interpretation of species as independent evolutionary lineages and is in line with the paradigm of integrative taxonomy (De Queiroz, 2007). The results of this study, based on four species delimitation discovery methods, such as ASAP, ABGD, GMYC and PTP, and two validation methods, as BPP and PHRAPL, revealed a high diversity within the genus *Apharyngostrigea* in Mexico. However, the DNA sequence-based approaches using nuclear and mitochondrial genes differed in the number of delimited species for the examined populations of *Apharyngostrigea*, particularly with GMYC and PTP, which relied on mitochondrial data. This is because species delimitation methods are based on different assumptions to distinguish evolutionary entities among individuals from different populations (Pons et al., 2006; Puillandre et al., 2012; Fujisawa and Barraclough, 2013; Miralles and Vences, 2013). In general, the analyses revealed a total of four nominal species (*A. cornu, A. pipientis, A. simplex* and *A. brasiliana*) and two candidate species and/or lineages within the genus with high posterior probability support values. Phylogenetic analyses based on nuclear and mitochondrial genes showed that *Apharyngostrigea* is monophyletic, coinciding with a recent study (see Locke et al., 2021). Additionally, molecular data confirm previous records of *A. pipientis* in the Americas (Dubois, 1968; Goldberg et al., 2002; Locke et al., 2021). Our specimens, collected from four ardeid hosts (*A. alba, N. nycticorax, B. virescens* and *Tigrisoma mexicanum* Swainson) across seven localities in Mexico, were nested within sequences morphologically identified as *A. pipientis* from Canada, the United States, Brazil and Argentina (Locke et al., 2021). They are also similar to sequences previously identified as *A. cornu* from the same hosts in Mexico, which were later reassigned to *A. pipientis* (Hernández-Mena et al., 2014; Locke et al., 2021). This digenean has also been recorded in other ardeid species in Africa and Korea, expanding its distribution range, possibly due to its broad host spectrum (Olivier, 1940; Pulis et al., 2011; Kim et al., 2020). We report for the first time the presence of *A. simplex* in southeastern Mexico. Our specimens collected from the Snowy Egret (*E. thula*) are morphologically similar to those reported by Ostrowski de Núñez, (1989) and Locke et al. (2021) from the same host in Argentina. However, *A. simplex* was originally described by Johnston (1904) from specimens found *E. novaehollandiae* and *E. garzetta* in Australia. Therefore, confirming the presence of this species in the Americas requires molecular comparison with sequences from Australian specimens. Finally, our specimens collected from the Great Blue Heron (*A. herodias*) in two localities in Mexico (Emiliano Zapata, Tabasco and Villa Tututepec, Oaxaca) clustered with sequences of adult specimens previously identified as *A. cornu* from the same host, as well as with metacercariae from three cyprinid species in North America (Locke et al., 2011). However, this species was originally described in Europe (Zeder, 1800) from the Great Heron (*A. cinerea*) and has been reported in several ardeid species in Africa and Central Asia (Richard, 1964; Dubois, 1968). Taxonomically, *A. cornu* exhibits considerable morphological variability, which may be attributed to its wide range of definitive hosts or could indicate a cryptic species complex. Therefore, sequences from European specimens are needed to clarify the status of *A. cornu* in the Americas (Locke et al., 2021).

Ukoli (1967) mentioned the difficulty in distinguishing species of *Apharyngostrigea,* based mainly on morphological characteristics such as the size and position of organs (e.g. ovary or testes). This variability depends on several factors, including the degree of parasite maturation, the physiological condition of the host, and the contraction or extension of the body during fixation, among others. In this sense, the taxonomic history of *Apharyngostrigea* is somewhat complex, as many species have been synonymized in various studies due to similar morphological characteristics, further complicating species delimitation (Dubois, 1938, 1968; Mishra and Gupta, 1975). In the present study, no distinct morphological differences were observed among the *Apharyngostrigea* species analysed. PCA did not reveal a clear separation between species of this genus, except for *A. brasiliana*, which formed a distinct cluster separate from the other species. This analysis also suggests that specimens labelled as Lineage 2 exhibit some characteristics similar to those of *A. cornu*, and others that bring it close to *A. simplex*. Further analyses focused on Lineage 2 will allow us to determine whether the observed features result from morphological variation within any of the previously described species for which molecular data are not yet available, or whether this lineage represents a yet undescribed species.

As recently suggested by Cribb et al. (2025), the recognition of trematode species remains a major ongoing challenge that must be addressed through an integrative approach. As many other genera, *Apharyngostrigea* includes some well-characterized species and others that are morphologically very similar, suggesting the presence of an unrecognized component of cryptic diversity. The use of molecular markers within an integrative taxonomy framework is crucial for species delimitation, particularly in cases where their taxonomic status is uncertain. The genus *Apharyngostrigea* currently contains approximately 20 species worldwide, of which six species are reported in the Americas (Ostrowski de Nuñez, 1989; Hernández-Mena, *et al.,* 2014; Locke et al., 2021; López-Jiménez et al., 2022). In this study, four discovery delimitation methods were implemented, such as ABGD, ASAP, GMYC and PTP and two validation methods, BPP and PHRAPL, in combination with morphological data. This study contributes to clarifying certain taxonomic hypotheses regarding species delimitation within this genus, reinforcing the taxonomic status of three species and identifying two lineages that may represent new species. However, further studies incorporating multiple lines of evidence are needed to fully assess the diversity of the genus and to elucidate the phylogenetic relationships among its species. The addition of other congeneric species of *Apharyngostrigea* from Europe, Africa and Asia Central is imperative to better understand the evolution of this group of digeneans.

## Supporting information

10.1017/S0031182025101315.sm001 López-Jiménez et al. supplementary material López-Jiménez et al. supplementary material

## Data Availability

Data will be made available on request.
